# Real-World Estimates of Adrenal Insufficiency–Related Adverse Events in Children With Congenital Adrenal Hyperplasia

**DOI:** 10.1210/clinem/dgaa694

**Published:** 2020-09-29

**Authors:** Salma R Ali, Jillian Bryce, Houra Haghpanahan, James D Lewsey, Li En Tan, Navoda Atapattu, Niels H Birkebaek, Oliver Blankenstein, Uta Neumann, Antonio Balsamo, Rita Ortolano, Walter Bonfig, Hedi L Claahsen-van der Grinten, Martine Cools, Eduardo Correa Costa, Feyza Darendeliler, Sukran Poyrazoglu, Heba Elsedfy, Martijn J J Finken, Christa E Fluck, Evelien Gevers, Márta Korbonits, Guilherme Guaragna-Filho, Tulay Guran, Ayla Guven, Sabine E Hannema, Claire Higham, Ieuan A Hughes, Rieko Tadokoro-Cuccaro, Ajay Thankamony, Violeta Iotova, Nils P Krone, Ruth Krone, Corina Lichiardopol, Andrea Luczay, Berenice B Mendonca, Tania A S S Bachega, Mirela C Miranda, Tatjana Milenkovic, Klaus Mohnike, Anna Nordenstrom, Silvia Einaudi, Hetty van der Kamp, Ana Vieites, Liat de Vries, Richard J M Ross, S Faisal Ahmed

**Affiliations:** 1 Developmental Endocrinology Research Group, School of Medicine, Dentistry & Nursing, University of Glasgow, Glasgow, UK; 2 Office for Rare Conditions, Royal Hospital for Children & Queen Elizabeth University Hospital, Glasgow, UK; 3 Health Economics and Health Technology Assessment, Institute of Health and Wellbeing, University of Glasgow, Glasgow, UK; 4 Lady Ridgeway Hospital, Colombo, Sri Lanka; 5 Department of Paediatrics, Aarhus University Hospital, Aarhus, Denmark; 6 Centre for Chronic Sick Children, Institute for Experimental Paediatric Endocrinology, Charité - Universitätsmedizin Berlin, Berlin, Germany; 7 Department of Medical and Surgical Sciences, Pediatric Unit, Center for Rare Endocrine Conditions (Endo-ERN), S.Orsola-Malpighi University Hospital, Bologna, Italy; 8 Department of Paediatrics, Technical University München, Munich, Germany; 38 Department of Paediatrics, Klinikum Wels-Grieskirchen, Wels, Austria; 9 Department of Paediatric Endocrinology, Amalia Childrens Hospital, Radboud University Medical Centre, Nijmegen, Netherlands; 10 University Hospital Ghent, Ghent University, Ghent, Belgium; 11 Pediatric Surgery Service, Hospital de Clínicas de Porto Alegre, UFRGS, Porto Alegre, Brazil; 12 Istanbul Faculty of Medicine, Department of Paediatrics, Paediatric Endocrinology Unit, Istanbul University, Istanbul, Turkey; 13 Department of Pediatrics, Ain Shams University, Cairo, Egypt; 14 Emma Children’s Hospital, Amsterdam UMC, Vrije Universiteit Amsterdam, Pediatric Endocrinology, Amsterdam, The Netherlands; 15 Pediatric Endocrinology, Diabetology and Metabolism, Department of Pediatrics and Department of BioMedical Research, Bern University Hospital Inselspital, University of Bern, Bern, Switzerland; 16 Department of Endocrinology, William Harvey Research Institute, Barts and the London School of Medicine, Queen Mary University of London, London, UK; 17 Department of Pediatrics, School of Medicine, Universidade Federal do Rio Grande do Sul (UFRGS), Porto Alegre, Brazil; 18 Marmara University, Department of Pediatric Endocrinology and Diabetes, Pendik, Istanbul, Turkey; 19 Health Science University, Medical Faculty, Zeynep Kamil Women and Children Hospital, Pediatric Endocrinology Clinic, Istanbul, Turkey; 20 Department of Paediatric Endocrinology, Sophia Children’s Hospital, Erasmus Medical Center, Rotterdam, The Netherlands; 21 Department of Paediatrics, Leiden University Medical Center, Leiden, The Netherlands; 22 Department of Endocrinology, Christie Hospital NHS Foundation Trust, Manchester, University Of Manchester, Manchester Academic Health Science Centre, Manchester, UK; 23 Department of Paediatrics, University of Cambridge, Cambridge, UK; 24 Department of Paediatrics, Medical University-Varna, UMHAT “Sv. Marina,” Varna, Bulgaria; 25 Department of Oncology and Metabolism, University of Sheffield, Sheffield, UK; 26 Birmingham Women’s & Children’s Hospital, Department for Endocrinology & Diabetes, Birmingham, UK; 27 Department of Endocrinology, University of Medicine and Pharmacy Craiova, University Emergency Hospital, Craiova, Romania; 28 Department of Paediatrics, Semmelweis University, Budapest, Hungary; 29 Unidade de Endocrinologia do Desenvolvimento, Laboratório de Hormônios e Genética Molecular/LIM42, Disciplina de Endocrinologia, Hospital Das Clinicas, Faculdade De Medicina, Universidade de Sao Paulo, São Paulo, Brazil; 30 Department of Endocrinology, Mother and Child Health Care Institute of Serbia “Dr Vukan Čupić,” Belgrade, Serbia; 31 University of Magdeburg, Magdeburg, Germany; 32 Karolinska University Hospital, Stockholm, Sweden; 33 Pediatric Endocrinology Regina Margherita Children’s Hospital, Città della Salute e della Scienza, University of Turin, Turin, Italy; 34 Wilhelmina Kinderziekenhuis, Division of Pediatric Endocrinology, Utrecht, Netherlands; 35 Centro de Investigaciones Endocrinológicas, División de Endocrinología, Hospital de Niños Ricardo Gutiérrez, Buenos Aires, Argentina; 36 The Jesse and Sara Lea Shafer Institute of Endocrinology and Diabetes, Schneider Children’s Medical Center of Israel, Petah Tikvah, Israel; 37 Sackler Faculty of Medicine, Tel Aviv University, Tel Aviv, Israel

**Keywords:** 21-hydroxylase deficiency, adrenal insufficiency, adrenal crisis, congenital adrenal hyperplasia, registry

## Abstract

**Background:**

Although congenital adrenal hyperplasia (CAH) is known to be associated with adrenal crises (AC), its association with patient- or clinician-reported sick day episodes (SDE) is less clear.

**Methods:**

Data on children with classic 21-hydroxylase deficiency CAH from 34 centers in 18 countries, of which 7 were Low or Middle Income Countries (LMIC) and 11 were High Income (HIC), were collected from the International CAH Registry and analyzed to examine the clinical factors associated with SDE and AC.

**Results:**

A total of 518 children—with a median of 11 children (range 1, 53) per center—had 5388 visits evaluated over a total of 2300 patient-years. The median number of AC and SDE per patient-year per center was 0 (0, 3) and 0.4 (0.0, 13.3), respectively. Of the 1544 SDE, an AC was reported in 62 (4%), with no fatalities. Infectious illness was the most frequent precipitating event, reported in 1105 (72%) and 29 (47%) of SDE and AC, respectively. On comparing cases from LMIC and HIC, the median SDE per patient-year was 0.75 (0, 13.3) vs 0.11 (0, 12.0) (*P* < 0.001), respectively, and the median AC per patient-year was 0 (0, 2.2) vs 0 (0, 3.0) (*P* = 0.43), respectively.

**Conclusions:**

The real-world data that are collected within the I-CAH Registry show wide variability in the reported occurrence of adrenal insufficiency–related adverse events. As these data become increasingly used as a clinical benchmark in CAH care, there is a need for further research to improve and standardize the definition of SDE.

Congenital adrenal hyperplasia (CAH) is an autosomal recessive condition with an incidence of approximately 1 in 15 000 and is most commonly due to 21-hydroxylase deficiency (1). In this form of CAH, a variable abnormality in steroidogenesis can result in varying levels of glucocorticoid (GC) and mineralocorticoid deficiency and in its most severe form, classic CAH, the patient may have severe primary adrenal insufficiency (2). CAH is the commonest cause of primary adrenal insufficiency in childhood (3), with a lifelong requirement for GC replacement and in most patients also mineralocorticoid replacement. Although GC treatment has improved survival in those with adrenal insufficiency (4), the risk of acute adrenal insufficiency, also known as an *adrenal crisis* (AC) has not been eliminated, with life expectancy remaining less than that of the general population (5, 6). An AC is usually associated with hospitalization, but the incidence of AC is very variable, with rates of between 5.2 and 10.9 per 100 person-years recently reported in children with CAH (7-10). It is possible that some of this variation is related to lack of a universally accepted definition of AC as well as its prodrome, often referred to as a *sick day episode* (SDE) (11, 12); it is also possible that there may be other factors that influence this variation, some that are patient-dependent and some that reflect on health care delivery (13). Nevertheless, there is increasing consensus that the avoidance of acute adverse events due to adrenal insufficiency is one of the most important outcomes that can be routinely measured (14, 15).

Given the rarity of CAH as well as AC in children, knowledge on the epidemiology of acute adrenal insufficiency–related adverse events, including SDE, in CAH is limited (7-10, 16, 17), with a paucity of data in large, multicenter, international cohorts. The International Congenital Adrenal Hyperplasia Registry (I-CAH) represents a valuable resource for evaluating outcomes in this rare condition (18). In this study, using data from the I-CAH Registry, we sought to investigate the occurrence of adverse events, including SDE and AC, in children with classic 21-hydroxylase deficiency CAH and examine the association of these outcomes with clinical variables.

## Methods

### Study population

All patients under the age of 18 years who were registered as having 21-hydroxylase deficiency CAH were identified from the I-CAH Registry (https://home.i-cah.org/) in July 2019. The I-CAH Registry is an international database of pseudonymized information on patients with CAH and is approved by the National Research Ethics Service in the United Kingdom as a research database of information that is collected as part of routine clinical care (18). The data within the registry are deposited by clinicians following informed consent from patients or guardians. Only those cases of 21-hydroxylase deficiency on GC with or without mineralocorticoid (fludrocortisone [FC]) were included. For the purpose of this study, the phenotypic classification into salt-wasting and simple virilizing CAH was based on concurrent treatment with FC.

### Clinical data collection

The I-CAH Registry collects data on acute adrenal insufficiency–related adverse events since a patient’s last clinic visit. Clinicians with cases meeting the inclusion criteria were approached to enter data for a minimum of 2 clinic visits per patient per year. SDE and AC were based on the clinical judgment of the reporting clinician. For each SDE, data were available on: the duration of SDE (number of days); any predisposing condition at the time of SDE (infectious illness, surgery, other, not known); management of each SDE, including sick day management of oral steroids (doubled, >doubled, not increased, not known) and the requirement for intramuscular hydrocortisone (HC) injection (yes, no, not known); the occurrence of AC (yes, no, not known); the need for health professional input (self-managed at home, health professional only, emergency room, admission to hospital, intensive care unit). Data were also gathered about GC and FC regimens (preparation, dose, frequency) at the time of clinic visits. HC-equivalent dose (ED) was calculated by multiplying prednisolone dose by 5 or dexamethasone dose by 80 (19, 20). Total GC dose (HC-equivalent) was converted from mg to mg per m^2^ using the Mosteller formula for calculation of body surface area (BSA) (21), or using predefined BSA values for weight as outlined in the British National Formulary for Children (22). GC and FC doses were categorized as low, normal, or high, based on the Endocrine Society clinical practice guidance for 21-hydroxylase deficiency CAH (2018) (1) as follows: GC low <10 mg/m^2^/day, normal 10 to 15 mg/m^2^/day, high >15 mg/m^2^/day; FC low <50 µg/day, normal 50 to 200 µg/day, high >200 µg/day.

### Statistical analysis

A longitudinal analysis of repeated measures reported from multiple visits was performed. The main outcome measures were occurrence of SDE, an increase in sick day oral GC dose, HC injection, AC, and hospitalization defined as attendance at the emergency room or admission to hospital. Multilevel logistic regressions were applied to examine the association of these outcome measures with clinical data obtained at each visit including age, sex, phenotype, GC, and FC dose. Accordingly, a 2-level multilevel regression model was built with individual patients (level 1) nested within centers (level 2). Random (intercept) effects for both levels were included in the modeling to allow variations between individuals and centers to be accounted for. The observed frequency of SDE and AC was determined as incidence rate, calculated as the number of SDE and AC divided by person-years. For assessment of geographical differences in the occurrence of SDE and AC, participating countries were categorized as those from a low or middle income country (LMIC) or from a high income country (HIC) as defined by the 2019 World Bank classification (23). Inter-group comparison for these variables was performed by the Mann-Whitney U test. The Fisher Exact test was performed to compare proportions in different groups. Results are reported as frequencies and percentages, median (with ranges) or odds ratios (OR) and 95% confidence intervals (CI). Data analysis was performed using R statistical software version 3.5.3 and Minitab version 18 statistical software (Minitab LLC, State College, PA, USA).

## Results

### Case selection

**Figure 1.**
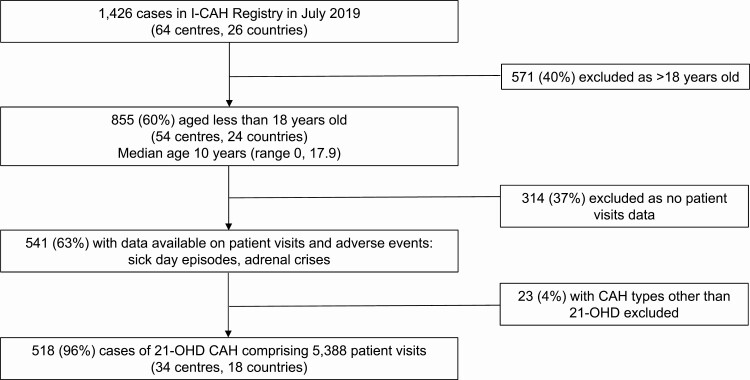
Participant selection from the I-CAH Registry. Abbreviations: 21-OHD, 21-hydroxylase deficiency; CAH, congenital adrenal hyperplasia; I-CAH, International Congenital Hyperplasia registry.

At the time of the study, 1426 cases of CAH were registered in the I-CAH Registry. Data on clinic visits including adverse events were available for 518 children younger than 18 years with 21-hydroxylase deficiency CAH (Fig. 1). There was no significant difference in the proportion of cases from HIC or LMIC countries, CAH type, or sex assigned between those with and without clinic visits data (24). Of the 518 children, 275 (53%) were girls, 459 (89%) had salt-wasting CAH and 59 (11%) had simple virilizing CAH. These children were reported from 34 centers in 18 countries with a median of 11 cases per center (1, 53) and 316 (61%) were from a high-income country (HIC). A total of 5388 clinic visits, occurring between 1984 and 2019, were evaluated in the 518 children, comprising a total of 2300 patient-years (Table 1). The median duration of follow-up per patient was 3 years (0.1, 17.9), with a median of 2.9 visits (0.3, 25.7) per patient-year. The median patient age at the time of the visits was 2 years (0, 17.9).

**Table 1.** Characteristics of Children With 21-Hydroxylase Congenital Adrenal Hyperplasia, at Clinic Visits (n = 5388)

	Group	Patients, n	Total visits, n (%)	Visits per patient, median (range)	Total follow-up duration (years)	Follow-up per patient (years), median (range)	Total visits per patient-year	Visits per patient-year, median (range)
Children, <18 y	All	518	5388 (total)	9 (1, 42)	2300	3.0 (0.1, 17.9)	1744	2.9 (0.3, 25.7)
Sex	F	275	2821 (52.4)	9 (1, 35)	1277	3.0 (0.1, 17.9)	881	2.9 (0.3, 18.2)
	M	243	2567 (47.6)	9 (1, 42)	1003	3.0 (0.2, 17.9)	863	3.0 (0.4, 25.7)
CAH phenotype	SW	459	5086 (94.4)	9 (1, 42)	1984	3.0 (0.1, 17.9)	1549	3.0 (0.3, 25.7)
	SV	59	302 (5.6)	9 (1, 35)	296	3.0 (0.2, 17.9)	195	2.5 (0.5, 14.7)
Age at visit, y	<1	418	1621 (30.1)	3 (1, 16)	234	0.7 (0.0,1.0)	2770	5.9 (2.3, 51.4)
	1–4.9	422	2427 (45.0)	5 (1, 18)	893	2.0 (0.2, 4.6)	1255	2.6 (0.6, 25.7)
	5–14.9	189	1147 (21.3)	5 (1, 26)	606	2.2 (0.0, 9.7)	578	2.7 (0.9, 22.5)
	15–17.9	61	193 (3.6)	3 (1, 12)	85	1.5 (0.2, 2.6)	170	2.3 (1.3, 10.4)
Number of SDE	0	512	4299 (79.8)	7 (1, 42)	2142	2.9 (0.0, 17.9)	1665	2.6 (0.3, 51.4)
	1	311	816 (15.1)	2 (1, 12)	667	0.9 (0.1, 17.6)	858	2.9 (0.2, 27.7)
	2–3	129	231 (4.3)	1 (1, 6)	165	0.3 (0.1, 14.0)	376	3.0 (0.1, 22.5)
	≥4	33	42 (0.8)	1 (1, 5)	17	0.3 (0.3, 2.7)	96	3.0 (0.8, 4.5)
Daily GC dose^*a*^	Normal	423	2019 (47.8)	4 (1, 24)	1224	1.8 (0.0, 17.2)	1508	2.7 (0.2, 53.3)
(HC ED mg/m^2^/d)	High	341	1201 (28.4)	3 (1, 17)	763	0.7 (0.0, 17.8)	1762	3.0 (0.2, 65.5)
	Low	287	1006 (23.8)	3 (1, 16)	496	0.9 (0.1, 17.2)	999	3.0 (0.3, 34.3)
	Not known	282	1162	3 (1, 20)	700	1.2 (0.0, 17.6)	861	3.0 (0.1, 30.0)
Daily FC dose^*a*^	Normal	466	4374 (90.2)	8 (1, 40)	1925	3.0 (0.1, 17.8)	1629	2.9 (0.2, 45.0)
(µg/d)	High	36	111 (2.3)	2 (1, 10)	23	0.3 (0.0, 2.7)	372	5.4 (1.2, 55.4)
	Low	95	359 (7.4)	2 (1, 16)	160	0.9 (0.2, 6.4)	283	3.0 (0.3, 8.7)
	Not known	143	544	2 (1, 25)	296	0.3 (0.0, 17.6)	695	3.0 (0.3, 65.5)

GC and FC doses were categorized as low, normal, or high, based on Endocrine Society clinical practice guidance for 21-hydroxylase deficiency CAH (2018) (1) as follows: GC low <10 mg/m^2^/day, normal 10 to 15 mg/m^2^/day, high >15 mg/m^2^/day; FC low <50 µg/day, normal 50-200 µg/day, high >200 µg/day.

Abbreviations: CAH, congenital adrenal hyperplasia; FC, fludrocortisone (mcg/day); GC, glucocorticoid; HC ED, hydrocortisone equivalent dose (mg/m^2^/day); SDE, sick day episodes.

^*a*^For GC and FC doses, the percentage of total visits was calculated based on “known” (Normal/High/Low) values.

### Steroid replacement

Of the 5388 clinic visits, information on steroid replacement was available in 5250 visits (97%). Of the 518 children, 435 (84%) were receiving HC, with 19 (3.5%) and 7 (1.5%) receiving prednisolone and dexamethasone, respectively; 16 children (3%) were taking a combination of different types of GC. HC use was documented in 4733 visits (90%) and the median steroid replacement dose, as HC ED, was 12.5 mg/m^2^/day (2.3, 71.4). In the 4226 visits where GC doses were known, the GC doses were reported to be within the recommended HC ED of 10 to 15 mg/m^2^/day in 2019 visits (48%) (Table 1). Of the 518 children, 459 (89%) were receiving FC at the time of clinic visits, with a median dose of 93.8 µg/day (12.5, 500) and doses were reported to be within the recommended range of 50 to 200 µg/day in 4374 visits (90%) (Table 1).

### Occurrence of sick day episodes and adrenal crises

**Figure 2.**
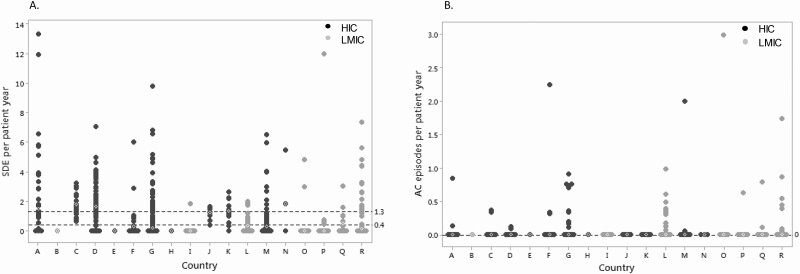
A. Sick day episodes (SDE) per patient-year per country. Countries labeled “A” to “R.” B. Adrenal crisis (AC) episodes per patient per country. Countries labeled “A” to “R.” Each point represents the median number of SDE or AC per patient-year for each patient in that country. The white diamond symbol indicates the median number of SDE or AC per patient-year for all patients in that specific country. The dashed horizontal black lines indicate the overall median (and 75th centile values for SDE) for SDE and AC per patient-year for all countries. Abbreviations: HIC, high income country; LMIC, low-middle income country.

Of the 5388 visits in 518 children, a total of 1544 SDE were reported in 1089 visits (20%) from 334 children (64%), of whom 167 (50%) were female. Based on a total observation period of 2300 patient-years, the SDE incidence rate was calculated at 67 per 100 patient-years and the median duration of a SDE was 3 days (1, 41). Of the 1089 visits where a SDE was reported, in 989 (91%), the reported number of SDE since last clinic visit was ≤2. A median of 1 SDE (1, 8) was reported per patient per visit, and the median number of SDE per patient-year per center was 0.4 (0, 13.3) (Fig. 2). The median SDE per patient-year was 0.75 (0, 13.3) vs 0.11 (0, 12.0); *P* < 0.001, in HIC and LMIC countries, respectively. Of the 1544 SDE, 62 events (4%) were associated with an AC in 49 out of 334 children (15%), all of whom had salt-wasting CAH and of whom 22 (45%) were female. The overall median AC per patient-year was 0 (0, 3), with no significant difference between HIC and LMIC countries, (0 [0, 2.2] vs 0 [0, 3.0]; *P* = 0.43), respectively (Fig. 2). There was no AC-related mortality reported among these children. The AC incidence rate in those children who had an SDE was 3.9 per 100 patient-years, with an overall AC incidence rate of 2.7 per 100 patient-years for all children. On examining temporal trends, from 1980 to 2000, 12 AC were reported in association with 37 SDEs in a total of 6 patients; thus, 32% of SDEs were associated with an AC. For the same 20-year period after 2000, 50 AC were reported in 1507 SDEs in a total of 43 patients; thus, only 3% of SDEs were associated with an AC following the year 2000.

### Risk factors for sick day episodes and adrenal crises

**Figure 3.**
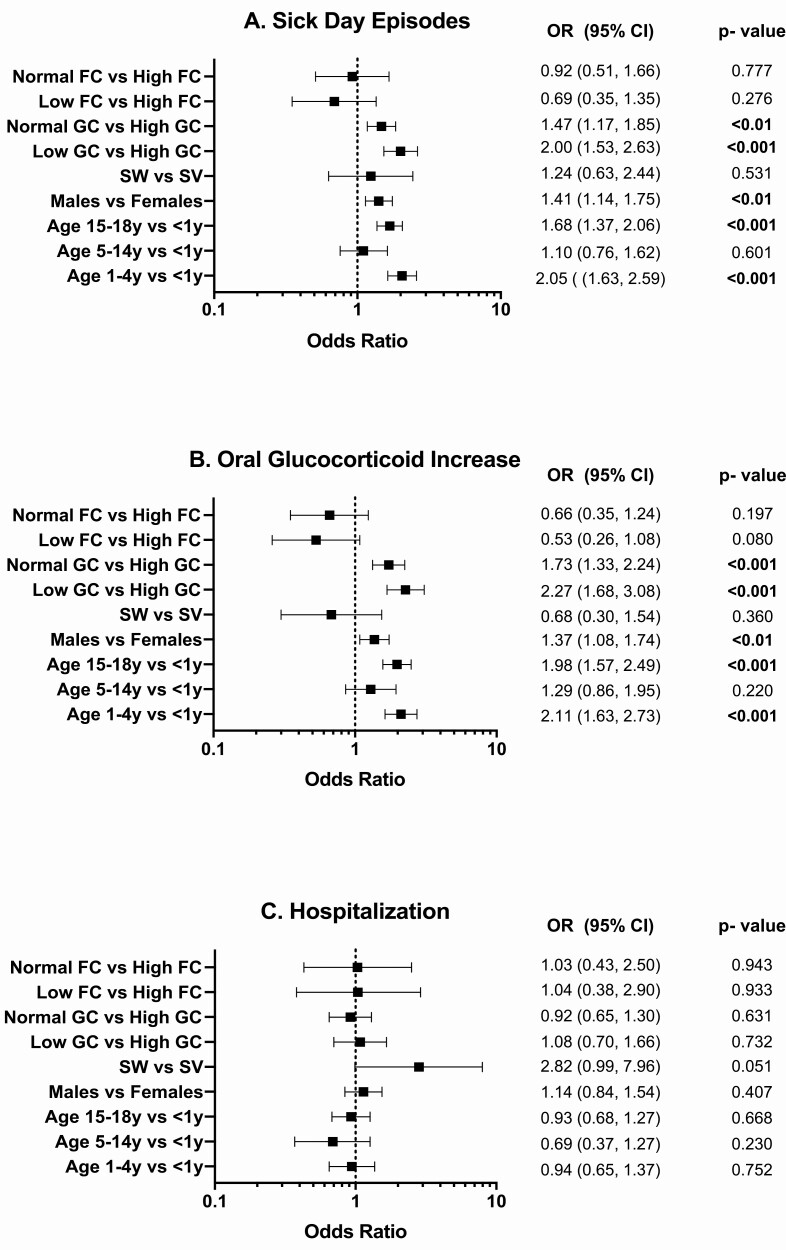
Association of age, sex phenotype, and medication dose with sick day episodes (A), an increase in sick day oral GC (B) and hospitalization (C). Abbreviations: FC, fludrocortisone (µg/day); GC, glucocorticoid (hydrocortisone-equivalent dose, mg/m^2^/day); SW, salt-wasting CAH; SV, simple virilizing CAH. Statistically significant *P* values are in bold type.

Infectious illness was the most frequent event associated with SDE and AC across all ages, reported in 1105 of 1544 SDE (72%) and in 29 of 62 AC episodes (47%). Surgery was reported as a factor predisposing to SDE in 58 (4%) episodes and no predisposing factor was specified in 381 SDE (24%) and 33 AC (53%). Younger children (aged 1-4 years) and adolescents (15-18 years) had a greater likelihood of a SDE (OR 2.05 [95% CI, 1.63-2.59] and OR 1.68 [95% CI, 1.37-2.06]; both *P* < 0.001, respectively) and an increase in sick day oral GC (OR 2.11 [95% CI, 1.63-2.73]; *P* < 0.001 and OR 1.98 [95% CI, 1.57-2.49]; *P* < 0.01, respectively) compared with children <1 year of age (Fig. 3). Compared to girls, boys were more likely to have SDE (OR 1.41 [95% CI, 1.14-1.75]; *P* < 0.01) and an increase in sick day oral GC (OR 1.37 [95% CI, 1.08-1.74]; *P* < 0.01). Children receiving lower GC doses (HC ED < 10 mg/m^2^/day) were more likely to have a SDE (OR 2.00 [95% CI, 1.53-2.63]; *P* < 0.001) and an increase in sick day oral GC (OR 2.27 [95% CI, 1.68-3.08]; *P* < 0.001) than those on higher GC doses (HC ED > 15 mg/m^2^/day). Similarly, children receiving GC doses within the recommended range (HC ED of 10-15mg/m^2^/day) were more likely to have SDE (OR 1.47 [95% CI, 1.17-1.85]; *P* < 0.01) and an increase in sick day oral GC (OR 1.73 [95% CI, 1.33-2.24]; *P* < 0.001), than children on higher GC doses (HC ED > 15 mg/m^2^/day). Due to the small number of AC events overall, which decrease further when broken down over risk factor levels, the multilevel regression model parameters could not be reliably estimated and are not reported.

### Management of adverse events

**Figure 4.**
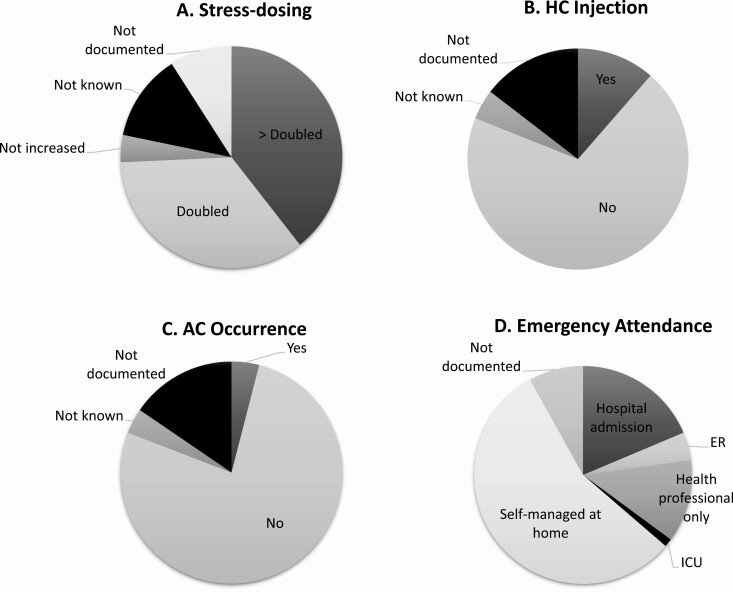
The management of sick day episodes (n = 1544). (A) Stress dosing, (B) HC injection, (C) AC occurrence, (D) Emergency attendance. Abbreviations: AC, adrenal crisis; ER, emergency room; HC, hydrocortisone; SDE, sick day episodes.

Of the 1544 reports of SDE, there was information on a change in the patient’s usual management documented in 1451 SDE (94%). More specifically, an increase in oral GC dose was noted in 1147 episodes (85%), HC injection was administered in 176 SDE (13%), and information on medical input was available in 1419 SDE (92%) (Fig. 4). A total of 858 SDE (60%) were self-managed in the community and of these, 6 (1%) were associated with an AC. In 561 SDE (40%), medical input, including hospitalization was sought and 55 (10%) of these were associated with an AC. In total, of the 62 episodes of AC that were reported, 55 (90%) were associated with hospitalization. An increase in oral GC or HC injection administration was reported in 52 (84%) episodes of AC; in the remaining 10 (16%) episodes of AC, there was no information regarding an increase in oral GC dose or the administration of HC injection; however, all 10 of these episodes were associated with hospitalization.

## Discussion

We report the largest multicenter, international cohort study to date evaluating the epidemiology of acute adrenal insufficiency–related adverse events in children with 21-hydroxylase deficiency CAH. While the majority of studies have measured AC, this is the first study to obtain a more complete perspective on adverse events by measuring SDE, episodes associated with sick day GC doses, and hospitalization in a global cohort of children.

Interestingly, the incidence rate of AC of 2.7 to 3.9 per 100 patient-years is at the lower end of the range that has been reported previously (7-10, 25). This is perhaps a reflection of improvements in parent/patient education and increased awareness of AC among families and clinicians. It is also useful to note that overall, less than 5% of SDE were associated with an AC. While it is possible that the management of SDE prevented an AC in these cases, confirmation of a causal link will require further study. Notably, all AC events occurred in children with the salt-wasting phenotype and there were no fatalities. In addition, a lower percentage of SDEs were associated with AC in the 2 decades after 2000 compared to previously; however, these temporal trends may not be reliable, as the data from the older cohort is more likely to suffer from recall bias. Therefore, there is a need to look at temporal trends in more detail and this will become easier to study as regular data reporting is sustained over the longer-term by participating centers. The SDE incidence rate of 68 per 100 patient-years also deserves attention and further exploration. While controversy remains about the definition of a SDE as well as its appropriate management (16, 26, 27), a change in the patient’s usual medical management, including an increase in oral GC, HC administration, hospitalization, or documentation of a predisposing factor was noted in 94% of SDE. SDE incident rates have not been reported previously; thus, it is unclear as to whether the currently reported SDE rate is particularly high or low. Patients and parents, themselves, may have a different definition of a SDE compared with their clinician and this could be explored in the future by comparing patient/parent reporting to clinician reporting, with the inclusion of more objective criteria for SDE (28).

Infectious illnesses were the commonest event associated with SDE and AC, reported in more than two-thirds of all SDE and almost half of all cases of AC, as reported in previous literature (7-9, 16, 29). The precipitating event in AC was unknown in half of all cases; it is likely that future studies incorporating prospective data collection may facilitate differentiation between specific etiology of infectious illness in children having SDE and AC. More recently, it has been speculated that patients with primary adrenal insufficiency, including CAH, may have a greater predisposition to infections (30) and these associations require further investigation.

Our data show wide variation in the median number of SDEs occurring per patient-year among centers. Moreover, more than half of patients with SDEs were self-managed at home but the majority of those who had an AC were hospitalized. A number of those who were hospitalized did not have an AC reported, thus raising the issue about the overlapping definitions of SDE and ACs. In the absence of a clearer distinction between an AC and a SDE, it is possible that hospitalization may be a better definition of a significant adverse event related to adrenal insufficiency. However, it is of course possible that different centers may have a different threshold for “hospitalizing” a patient with an adrenal insufficiency–related adverse event. This threshold for hospitalization may depend on several factors including health care delivery models, health care utilization costs, patient education, as well as the perceived competence of the patient in self-managing their condition (9, 31, 32). In addition, patients from HIC countries had a higher median SDE per patient-year compared with those from LMIC countries. One may expect that patients from LMIC and more deprived communities would have greater SDEs and ACs, due to issues such as reduced access to hospital care and medication. Thus, the factors that influence these results require further evaluation.

The optimal management of children with CAH requires GC regimens that achieve a balance between normal growth and development while avoiding hyperandrogenism and the risk of adverse events associated with adrenal insufficiency (1). Consistent with recommendations from international guidelines, children were reported to be receiving maintenance GC within the recommended range in almost half of clinic visits, with FC doses within recommended dose ranges in 90% of visits. In the current study we observed that the likelihood of SDEs was lowest in those who were on high doses of GC; future studies should also compare the health economic cost of the morbidity associated with high GC doses against that of adrenal insufficiency–related adverse events. The dose of 10 to 15 mg/m^2^/day used in children with CAH is greater than that generally recommended for other forms of adrenal insufficiency (33), although there are scarce data on doses of GC used in children apart from in CAH. Furthermore, the majority of patients in our study were very young children and it is recognized that children with CAH may have differing requirements for GC throughout childhood (19); thus, further studies in larger cohorts of children of all ages are required.

A greater likelihood of SDE and sick day dosing was also observed in boys compared with girls. This is contrary to the findings of others where these adverse events were more likely in girls (7, 34). While it is clear that newborn boys with CAH are more likely to present with a salt-wasting crisis when the diagnosis is delayed, it is unclear why boys, or for that matter, girls on maintenance therapy would have a higher incidence of adrenal insufficiency–related adverse events. The majority of studies in adult populations have not revealed a gender difference, although one study reported a higher incidence of adverse events in women with secondary adrenal insufficiency (35) and another reported a greater incidence in men with concomitant hypogonadism (36). Interestingly, children with a salt-wasting phenotype comprised 89% of cases of 21-hydroxylase deficiency in our cohort. This is higher than that previously reported in the literature (2) and may be attributed to several factors including the complex mix of cases being entered by international clinicians, for example, in countries without newborn screening, such as the UK, affected boys may be missed or diagnosed with simple virilizing CAH much later when signs of androgen excess develop, which may perhaps lead to delayed entry into disease specific registries. The current study also showed that younger (1-4 years) and older (15-18 years) age groups had the greatest risk of SDE. A higher rate of illness episodes in childhood and lower rates of administration of HC injection among very young children have been previously reported as reasons for this observation (7, 16), with young age also consistently recognized as a risk factor for AC (28, 37). These findings may reflect parental uncertainty or inexperience with regards to management of illness episodes in children (38, 39). A higher rate of SDE in adolescents may be attributed to the period of transition from pediatric to adult services, greater patient autonomy, and reduced adherence to therapy (9, 40, 41).

It is possible that this study may experience clinician and patient retrospective recall bias with subjective reporting and a potential for under- or over-reporting of SDE and AC. There may have also been a degree of selection bias, as not every patient at a center had been included in the I-CAH Registry and among those who had been included in this registry, a variable number of cases had a sufficient amount of data to be included in the study. The lack of a universal definition for “sick day episodes” can be considered a limitation. The definition of SDE used herein was based on self-reported physician diagnosis and clinical judgment of the reporting clinician of these events. The current observations highlight the limitation of this definition by not showing any value of SDEs for predicting AC. A combination of the variable criteria for defining SDEs as well as a variable threshold for reporting these adverse events may be an explanation for the observed differences. For instance, the higher prevalence of SDEs in children from HIC could be attributed to greater vigilance and reporting of minor illnesses among parents and clinicians from higher-income countries. Interestingly, there was no difference in the number of AC events between patients from HIC and LMIC, suggesting an equally effective level of illness management and AC prevention. Due to the small number of AC events overall, further studies in a larger cohort of patients with more patient visits and a greater number of ACs are required to investigate factors that may influence differences in the prevalence of ACs as well as SDEs. A better definition of SDE may be any episode that requires an increase in glucocorticoid replacement above the routine replacement dose, as per local sick day rules and to avoid an adrenal crisis. However, there is a need for further research to achieve a more effective definition of SDE that provides real clinical significance. Furthermore, an online system for collecting patient-reported accounts of SDE and their management may also prove useful in understanding the variation between centers. Lastly, the study did not attempt to assess clinical variables such as the biochemical adequacy of steroid replacement (42, 43). On the other hand, the structured manner of real-world data collection within the I-CAH Registry, the size of the cohort and its potential to represent global practice with the availability of adverse events data for more than half of registered patients under the age of 18 years were clear strengths. Although, the investigators did not have recourse to source data, the data that have been collected have previously been reported to have a high degree of validity, consistency and accuracy (44).

In summary, the real-world data in the I-CAH Registry can be used to identify benchmarks for a range of adrenal insufficiency–related acute adverse events which seem to show clear variation between centers. The current study provides the framework that can be used for studying the effect of targeted interventions that are aimed at improving the care of children with CAH.

## Data Availability

Some or all datasets generated during and/or analyzed during the current study are not publicly available but are available from the corresponding author on reasonable request.

## Abbreviations

AC: adrenal crisis
CAH: congenital adrenal hyperplasia
CI: confidence interval
ED: equivalent dose
FC: fludrocortisone
GC: glucocorticoid
HC: hydrocortisone
HIC: High Income Country
I-CAH: International Congenital Adrenal Hyperplasia Registry
LMIC: Low or Middle Income Country
OR: odds ratio
SDE: sick day episode

## Additional Information

***Disclosure Summary:*** R.J.M.R. is a Director of Diurnal Ltd.
